# Supplementary material for: Hybrid Models and Biological Model Reduction with PyDSTool
Source: PLoS Comput Biol. 2012 Aug 9;8(8):e1002628. doi: 10.1371/journal.pcbi.1002628 (PMC3415397; doi:10.1371/journal.pcbi.1002628)
Supplement: Text S4 — Complete source code for the PyDSTool package (version 0.88.120504). Includes API documentation and help files linking to web pages. This file is identical to the current public release on Sourceforge.net. (ZIP) [file pcbi.1002628.s004.zip › PyDSTool/html/toc-PyDSTool.ModelConstructor'-module.html]

xml version="1.0" encoding="ascii"?


ModelConstructor'


# Module ModelConstructor'

---

## Classes

Descriptor

EvMapping  
 GDescriptor  
 GeneratorConstructor  
 MDescriptor  
 ModelConstructor  

## Functions

embed  
 makeEvMapping  
 makeModelInfo  
 makeModelInfoEntry  

processReused

validateTransitionName

## Variables

API

Continuous

Discrete

Inf

LargestInt32

NAMESEP

NaN

\_all\_complex

\_all\_float

\_all\_int

\_all\_numpy\_complex

\_all\_numpy\_float

\_all\_numpy\_int

\_complex\_types

\_float\_types

\_int\_types

\_num\_equivtype

\_num\_maxmin

\_num\_name2equivtypes

\_num\_name2type

\_num\_type2name

\_num\_types

\_pytypefromtype

\_real\_types

\_seq\_types

isfinite

mathNameMap

null\_predicate

targetLangs

---

[hide private]
